# High-Throughput Sequencing of Small RNA Transcriptomes in Maize Kernel Identifies miRNAs Involved in Embryo and Endosperm Development

**DOI:** 10.3390/genes8120385

**Published:** 2017-12-14

**Authors:** Lijuan Xing, Ming Zhu, Min Zhang, Wenzong Li, Haiyang Jiang, Junjie Zou, Lei Wang, Miaoyun Xu

**Affiliations:** 1 Biotechnology Research Institute, The National Key Facility for Crop Gene Resources and Genetic Improvement, Chinese Academy of Agricultural Sciences, Beijing 100081, China; xinglijuan3@163.com (L.X.); lyanzhu0@126.com (M.Z.); 13126532159@163.com (M.Z.); lwzm1010@163.com (W.L.); zoujunjie@caas.cn (J.Z.); 2 School of Life Sciences, Anhui Agricultural University, Hefei 230036, China; jhy19800802@126.com

**Keywords:** maize (*Zea mays*), microRNA, differential expression, embryo, endosperm, kernel development, miR169

## Abstract

Maize kernel development is a complex biological process that involves the temporal and spatial expression of many genes and fine gene regulation at a transcriptional and post-transcriptional level, and microRNAs (miRNAs) play vital roles during this process. To gain insight into miRNA-mediated regulation of maize kernel development, a deep-sequencing technique was used to investigate the dynamic expression of miRNAs in the embryo and endosperm at three developmental stages in B73. By miRNA transcriptomic analysis, we characterized 132 known miRNAs and six novel miRNAs in developing maize kernel, among which, 15 and 14 miRNAs were commonly differentially expressed between the embryo and endosperm at 9 days after pollination (DAP), 15 DAP and 20 DAP respectively. Conserved miRNA families such as miR159, miR160, miR166, miR390, miR319, miR528 and miR529 were highly expressed in developing embryos; miR164, miR171, miR393 and miR2118 were highly expressed in developing endosperm. Genes targeted by those highly expressed miRNAs were found to be largely related to a regulation category, including the transcription, macromolecule biosynthetic and metabolic process in the embryo as well as the vitamin biosynthetic and metabolic process in the endosperm. Quantitative reverse transcription-PCR (qRT-PCR) analysis showed that these miRNAs displayed a negative correlation with the levels of their corresponding target genes. Importantly, our findings revealed that members of the miR169 family were highly and dynamically expressed in the developing kernel, which will help to exploit new players functioning in maize kernel development.

## 1. Introduction

Seed development is an important developmental process during a plant’s life cycle. Maize is a typical monocot and single-seeded plant. Maize kernel is comprised of a precocious embryo, persistent endosperm and surrounding maternal tissue-pericarp. The embryo, endosperm and pericarp undergo fine and strict regulation that can be divided into three phases: early development, reserve filling and dehydration [1]. Nuclear divisions occur without cellularization in the endosperm during the first 4 days after pollination (DAP), then the endosperm differentiates into four main cell types at about 6 DAP: the starchy endosperm (SE), the basal endosperm transfer layer (BETL), the aleurone layer (AL) and the embryo-surrounding region (ESR) [2,3]. At about 15 DAP, ESR disappears and synthesis of endosperm starch and storage proteins peak, then the endosperm enters the reserve filling stage. Starchy endosperm cell expansion terminates at around 20–25 DAP. Meanwhile, the embryo starts to differentiate into specified cell types: the shoot apical meristem (SAM) and the root apical meristem (RAM), while protective structures, the coleopile and the coleprhiza are formed around them from 9 DAP to 12 DAP [4].

Temporal and spatial expression and accumulation of messenger RNAs (mRNAs) during maize kernel development suggested specific regulation of gene sets [5,6]. An important mechanism controlling gene expression is executed by microRNAs (miRNAs). miRNA is a type of short non-coding RNA of approximately 21 nucleotides in length that can target mRNAs for cleavage or translational inhibition of gene expression at the post-transcriptional level. In recent years, many studies have shown that miRNAs play vital roles in almost all aspects of the biological processes associated with growth, development and stress response in plants [7]. A total of 2560 mature miRNAs encoded by 2063 precursors have been identified through computational or experimental methods from cereal crops including maize [8]. Sixty-five miRNAs have been experimentally validated through genetic analysis in major crops and about 66% of the targets of these miRNAs are transcription factors (TFs), indicating the “core” role of miRNAs in gene regulatory networks [9]. An increasing number of reports have shown that miRNAs play crucial roles in kernel development. Expression profiles of miRNAs in rice development were analyzed through miRNA microarray [10] and the expressed small RNAs (sRNAs) in superior and inferior spikelets at five distinct grain developmental stages were performed by high-throughput sequencing [11]. Furthermore, miRNAs have been proved to play important roles in grain development or grain filling in rice. SQUAMOSA-promoter binding protein-like (SPL) TFs are negatively regulated by miR156, which function in the regulation of grain size, grain quality and grain yield in rice [12,13,14]. miR396 can target growth-regulating factors (GRFs), which are involved in the control of grain size and yield in rice [15,16,17,18]. The laccase-like protein (LAC) was tuned by miR397, which affects grain size, grain number and yield in rice [19]. The expression of the APETALA2 (AP2) family of TFs can be dampened by miR172, which affects grain density in barley [20]. SPL TFs were antagonized by miR156 and miR157, which function in the regulation of vegetative and reproductive development and fruit ripening in tomato, respectively [21,22,23]. All the above results indicate that miRNAs are important for plant kernel development.

Many maize miRNAs have been identified through small RNA sequencing using samples of drought or salt-stressed kernellings, phosphate-deficient root and leaf, and sucrose-nursed endosperm [24,25,26,27]. Nevertheless, information on the dynamic expression profile and global miRNA/target modules regulation network throughout maize kernel development remains limited. The purpose of this study was to discover embryo- or endosperm-specific miRNAs and their potential targets and provide vital clues for further and detailed functional studies of maize miRNAs. In this study, a comprehensive miRNAomic study of maize embryo and endosperm at three key time points (9 DAP, 15 DAP and 20 DAP) through the first two development stages was conducted using high-throughput sequencing. Our results suggested an important role of miRNA networks and their expression levels in determining the maize kernel development. Studies on the miR169 family members revealed that they were highly expressed in the developing kernel, helping to exploit new players functioning in maize kernel development. Elucidation of the genetic regulatory mechanisms involved in maize kernel development will facilitate the design of strategies to improve yield and quality, and provide insight that is applicable to other monocotyledon plants.

## 2. Materials and Methods

### 2.1. sRNA Isolation, Library Preparation and Sequencing

The maize (*Zea mays*) inbred line B73 was grown at Wanzhuang Agricultural Research Station of Chinese academy of agriculture sciences (CAAS) during the 2015 growing season. Developing embryo and endosperm were collected 9 days after pollination (DAP), 15 DAP and 20 DAP, frozen immediately in liquid nitrogen, and stored at −80 °C until RNA extractions. Self-pollination and samples collection were performed as described previously [28]. Total RNA isolation and quality detection were performed as described previously [28]. Starting from the total RNA samples, sRNAs were size selected and ligated for sequencing following the TruSeq Small Prep Kit Preparation (Illumina, San Diego, CA, USA). Illumina sequencing was performed by OE Biotech Co., Ltd. (Shanghai, China). sRNA raw sequencing data are available from the National Center for Biotechnology Information Sequence Read Archive [29] with accession numbers SRX3203296, SRX3203297, SRX3203298, SRX3203299, SRX3203300, SRX3203301.

### 2.2. sRNA Bioinformatic Analysis

The quality of RNA-Seq reads was examined using cutadapt 1.14 [30]. The quality control step included removal of reads (>41 and <20 base pair) and trimming of reads containing adapter/primer contamination. Low quality (>80% of Q20) reads were removed by fastx-toolkit [31], followed by a screening of reads that included N by NGS QC Toolkit [32]. After quality control, redundant reads were removed to retain only the unique reads by fastx-toolkit [31], and the read count for each sequence was recorded. Only the reads with length 18–41 nt were retained for further analysis.

Before processing the data for miRNA prediction, all the filtered unique reads from each sample were matched against the miRbase database [8]. Perfectly matched sequences were known miRNA and the expressional level was calculated by bowtie software [33]. The remaining unmatched sequences were blasted against annotated non-coding RNA sequences, including plant snoRNA (Plant SnoRNAbase v1.2; [34], transfer RNA (tRNA) [35] and ribosomal RNA (rRNA) (RFAM, v11.0) [36] etc., using blast [37]. The remaining reads were screened against degradated transcripts using the bowtie2 software [38] and repeat sequences from RepBase [39] using RepeatMasker [40]. The reads that were mapped onto these database sequences were discarded. The retained 18–41 nt reads were mapped onto the B73 reference genome (V3) using the bowtie2 software [38]. All perfectly matched sRNAs were retained for miRNA prediction. Finally, secondary structure prediction of individual miRNAs was performed with the RNAfold software [41] using the default folding conditions. The mature miRNA-5p and -3p candidates were identified and quantified based on their read counts. The parameters of used software were set to default.

### 2.3. miRNA Gene Expression Analysis and Calculation of the Fold-Changes and p-Values

To compare the differentially expressed miRNAs and the expressional patterns between the embryo and endosperm at the same time point or among different developmental stages in the same tissue during kernel development, the abundance of each miRNA in the six libraries was normalized to transcripts per million (TPM). Expression levels lower than 0.01 TPM were declared unexpressed miRNAs. Calculation of the fold-changes and *p*-values from the normalized expressions was performed as previously described [42].

### 2.4. Prediction of miRNA Targets and Functional Annotation

Putative targets of the differential miRNAs were predicted with psRNATarget [43]. The maize genome annotation [44] was used to find the putative function of the predicted targets. Gene Ontology (GO) enrichment analysis was performed with agriGO program [45] with *p*-value cut-off of ≤0.05. *Zea mays* locus ID v3.30 (Gramene Release 50) was used as background or reference. The background showed the percentage of genes involved in a certain function, against the whole genome.

### 2.5. Expression Validation of miRNA and Their Targets

To validate the miRNA gene expression results, quantitative reverse transcription-polymerase chain reaction (qRT-PCR) analysis was performed. Firstly, sRNAs were isolated and reverse-transcribed using a miRcute miRNA Isolation Kit (TIANGEN, Beijing, China) and miRcute Plus miRNA First-Strand cDNA Synthesis Kit (TIANGEN, Beijing, China) according to the manufacturer’s instructions. Then, we performed qPCR reactions using an ABI7500 real-time system (Applied Biosystems, Foster City, CA, USA) and miRcute miRNA qPCR Detection Kit (TIANGEN, Beijing, China) using the following parameters: 1 min at 95 °C, 40 cycles of 15 s at 95 °C, 1 min at 60 °C, followed by the melt curve phase. The expression of U6 small nuclear RNA (snRNA) was used as an internal control to normalize for variance in the quantity of miRNA. Three independent biological replicates of each sample and three technical replicates of each biological replicate were included in qRT-PCR analysis. The mean cycle threshold (CT) value (from three technical replicates) of each miRNA was normalized to the mean CT value of U6 for individual tissue samples. For each biological replicate, the relative expression level of each miRNA in different tissue samples was calculated using the standard delta delta CT method. The average expression levels from three biological replicates and standard deviation were calculated for each tissue sample. Validation of target expression was performed as described previously [28]. Primer sets for the selected miRNAs and mRNAs are listed in Table S1.

### 2.6. Histological Observation of Transgenic Kernel

For PromiR169: GUS (β-glucuronidase) constructs, 2000-bp upstream regions of the precursors of zma-miR169b, zma-miR169c and zma-miR169i amplified from genomic DNA (all primer sequences used for cloning are listed in Table S1), were cloned into pEASY-T1 vector (TransGen Biotech, Beijing, China) and then were recombined into the binary vector pCAMBIA3301 using ClonExpress^®^ II One Step Cloning kit (Vazyme Biotech Co., Ltd., Nanjing, China) after sequencing confirmation. HiII transformation was performed by Beijing Plantgm Biological Technology Development Co., Ltd. (Beijing, China) and independent stable transgenic lines were selected for further analysis. The histochemical detection of GUS activity in kernels was performed with GUS staining kit (Real-Times Biotechnology Co., Ltd., Beijing, China). Then, the samples were destained by 75% ethanol and the images were obtained using microscope M165 FC (Leica, Wetzlar, Germany).

## 3. Results

### 3.1. Overview of sRNAs Expressed in Maize Embryo and Endosperm during Kernel Development

**Figure 1:**
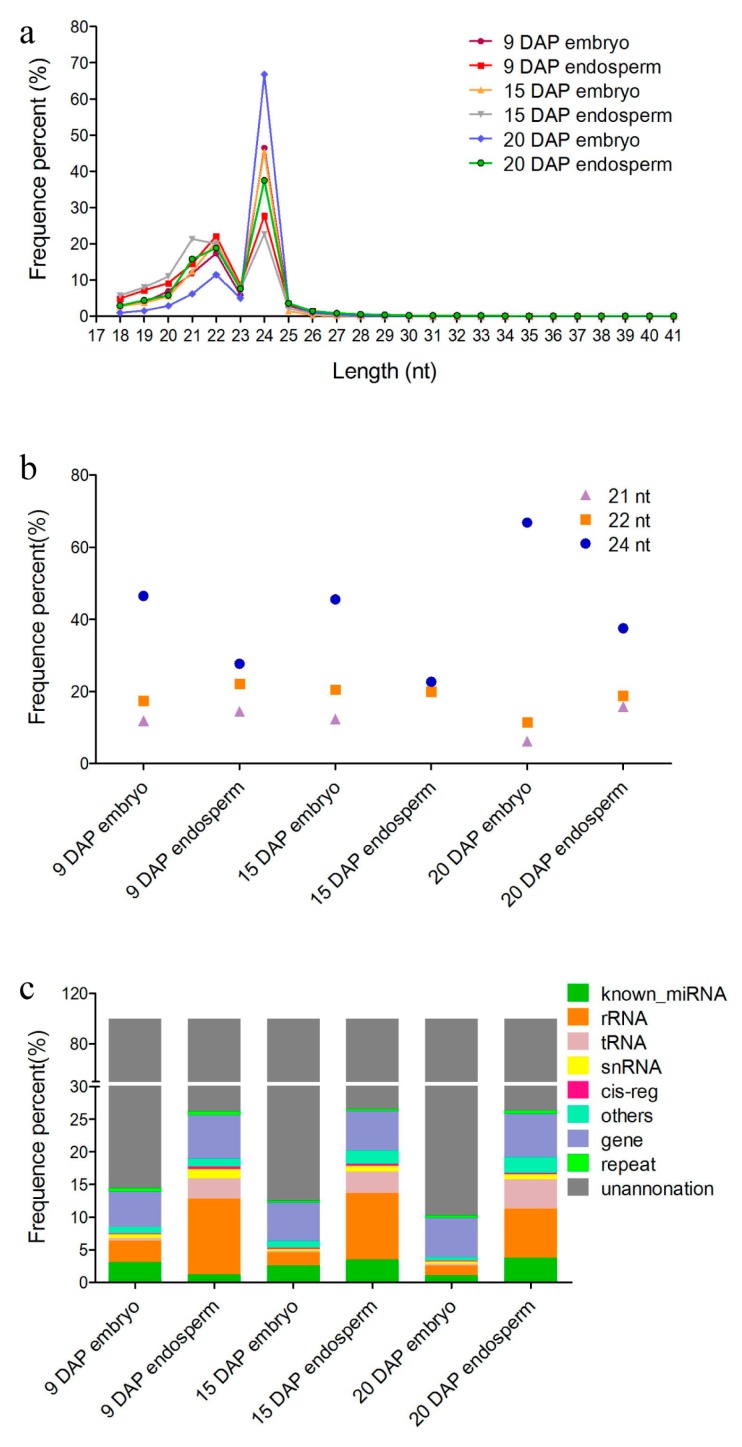
Overall small RNAs (sRNAs) dynamics during the maize kernel development process. (**a**) Line charts showing the length-distribution of total sRNAs from six deep sequenced libraries at different maize kernel development stages. (**b**) Dot chart showing different percentages of the 24-nt, 22-nt and 21-nt total sRNAs presenting in the embryo and endosperm at different kernel development stages. (**c**) Bar graph showing the sRNA dynamics of different categories from the six libraries of different tissues and development stages. rRNA: ribosomal RNA; tRNA: transfer RNA; snRNA: small nuclear RNA.

**Figure 2:**
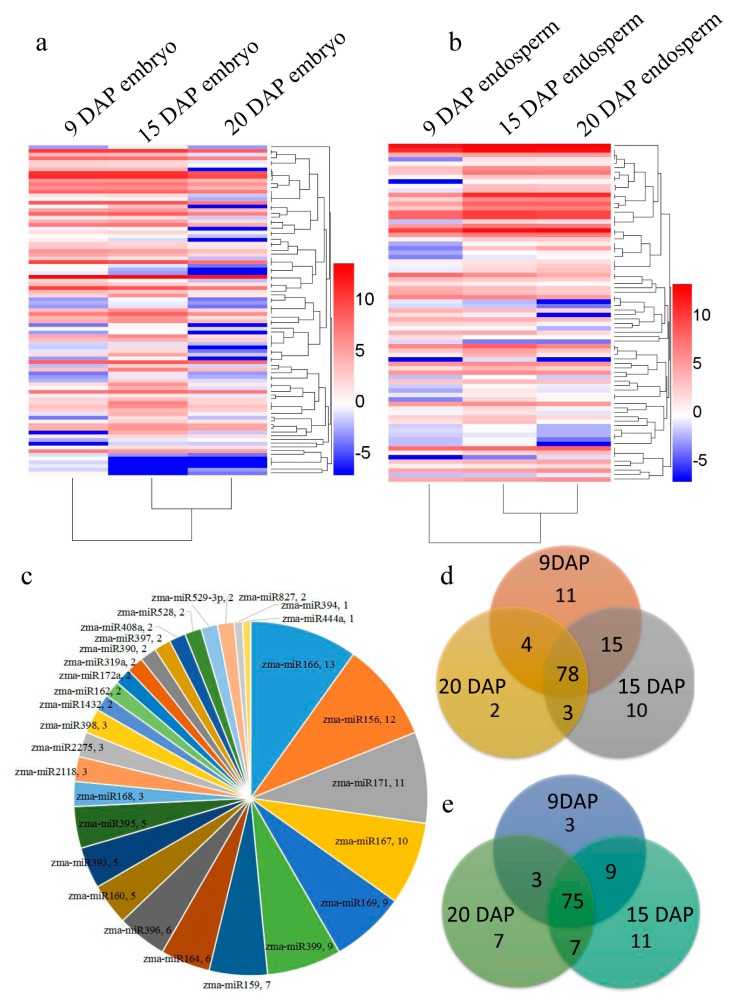
Expression profiles of microRNAs (miRNAs) during embryo and endosperm development. (**a**) The cluster heatmap of all miRNAs’ expression at different stages during embryo development; (**b**) The cluster heatmap of all miRNAs’ expression at different stages during endosperm development; (**c**) miRNA family distribution of total kernel miRNA; digits mean the number of expressed mature miRNA members; (**d**,**e**) Venn diagram showing common and specific miRNAs among three samples; d is embryo; e is endosperm. DAP: days after pollination.

To obtain an overview of the miRNA expression profile in the embryo and endosperm during maize kernel development, sRNA libraries were constructed using samples from three different developmental stages: 9 DAP, 15 DAP and 20 DAP. After removal of low-quality and adaptor contaminants (reads <18 nt and >41 nt) from the raw sequencing data, a total of 11,416,565; 9,157,084; 11,820,583; 10,275,288; 11,788,293 and 11,797,076 clean reads were obtained from the libraries of 9 DAP embryo, 9 DAP endosperm, 15 DAP embryo, 15 DAP endosperm, 20 DAP embryo and 20 DAP endosperm, respectively. There were three dominant populations of sRNAs in all cases according to their lengths (Figure 1a,b). The concentrated length distribution with the peak at 24 nt accounting for 46.49%, 27.7%, 45.5%, 22.67%, 66.81% and 37.48% in the six libraries respectively indicated that Small interfere (siRNA)-associated regulation with heterochromatin modification and RNA-directed DNA methylation [46] is involved in maize kernel development. This is also consistent with previous studies in rice and barley which showed that 24-nt sRNAs were most abundant in filling or developing grains [11,47]. The 22-nt sRNAs were the second most abundant population present in six libraries, representing 17.39%, 22.12%, 20.55%, 19.99%, 11.42% and 18.84% of the total reads, respectively (Figure 1a,b). Furthermore, there were more 24-nt sRNAs present in the embryo than in the endosperm at all three kernel development stages investigated, whereas the 22-nt sRNAs showed a reversed trend (Figure 2b), which may be due to tissue- and temporal-specific expressions of sRNAs during maize kernel development. The 21-nt small RNAs were the third most abundant, accounting for 11.86%, 14.47%, 12.35%, 21.31%, 6.14% and 15.76% of the total reads in six libraries, respectively (Figure 1b). After discarding reads which could be annotated as rRNA, repeats, exons or introns (Figure 1c), the remaining reads were used for miRNA prediction and analysis. We observed a higher percentage of total known miRNA that was expressed in the endosperm than in the embryo during three maize development stages (Figure 1b). Other types of sRNAs that were characterized for each dataset were mainly associated with rRNAs, tRNAs and snRNAs. These sRNA populations contained more diverse sRNAs with more reads in the endosperm than in the embryo at all development stages (Figure 1c).

### 3.2. Identification of Known and Novel miRNAs Expressed in Maize Embryo and Endosperm

Clean sRNA reads were aligned to the miRNA precursor/mature miRNA of plant and animals deposited in miRBase 21.0 [8]. A total of 132 known miRNAs from our library matched miRBase (Table S2). Analysis in terms of TPM read counts for known miRNA families indicated that the expression frequency varied significantly among different miRNA families.

To reveal the novel miRNA candidates, we explored the characteristic hairpin structure of miRNA precursors by using miRdeep2 [48]. Only secondary structures with the lowest free energy and a high degree of pairing were defined as miRNA precursors. Six novel miRNAs and their precursors (Table S3, Figure S1) were predicted. The read counts of the novel miRNAs ranged from 18 to 193. The length of precursors of the novel miRNAs ranged from 52 nt to 81 nt. The stem loop structures of predicted novel miRNA candidates were taken from the precursor sequences by using RNAFold [41] (Figure S1). The whole expression profile of the above 138 miRNAs (132 known and 6 novel) is presented in Figure 2a,b, and more miRNAs were expressed in 15 DAP embryos and endosperm. A total of 132 known miRNAs belonged to 28 families. Six miRNA families zma-miR166, zma-miR156, zma-miR171, zma-miR167, zma-miR169 and zma-miR399 were predominantly expressed in maize kernel (Figure 2c, Table S2). In total, 13, 12, 11, 10, 9 and 9 miRNA members were included in these families respectively, close to fifty percent of total kernel-miRNA (Figure 2c). As shown in the Venn diagram, 84 miRNA families in the embryo and 81 miRNA families in the endosperm were detected in the developing kernels throughout the three stages (Figure 2d,e).

**Figure 3:**
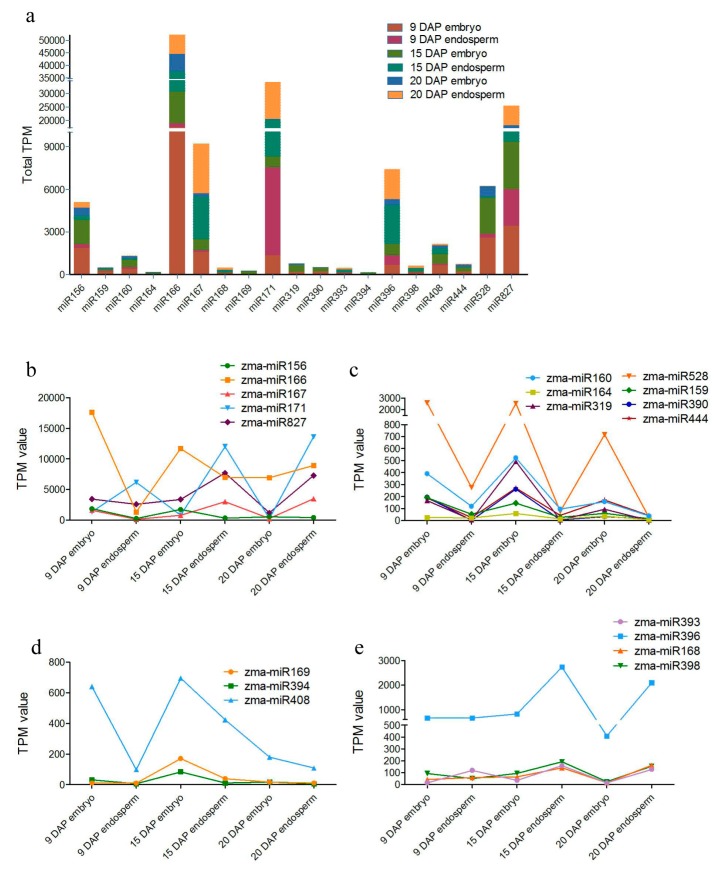
Summary of highly expressed and kernel-specific miRNAs in the maize embryo and endosperm. The total number of sequences from six libraries is presented in terms of transcript per million (TPM). (**a**) Distributions of highly expressed miRNA. (**b**–**e**) The expressional patterns of highly expressed miRNAs.

The selected known miRNA families with more than 50 TPM reads were classified into 19 families (Figure 3a and Table S4). Among them, miR166 was the most abundant family (17,602) followed by miR171 (11,988), miR827 (7686), miR167, miR396, miR528, miR156, miR408, miR160, miR390, miR159, miR444, miR319, miR398, miR168, miR394, miR164, miR393 and miR169 (Figure 3a). These miRNAs showed variation in their expression at different stages (Table S4). Each miRNA family featured various counts with its own variants. For instance, miR169o was highly expressed in 15 DAP kernel, and was significantly higher expressed in the embryo compared to the endosperm (Figure 3a; Table S4). With these expression data, we can look into how these kernel-abundant miRNAs are involved in embryo and endosperm development. Kernel-abundant miRNAs were divided into four groups according to their expression profile (Figure 3b). Expression levels of miR156, miR166, miR167, miR171 and miR827 decreased with the development of the embryo, but increased with the development of the endosperm, indicating that their functions are potentially involved in the switch from embryo to endosperm development (Figure 3b). MiR159, miR160, miR164, miR319, miR390 and miR444 were expressed mainly in the embryo at 9 DAP and 15 DAP, showing their regulatory function in early embryo development (Figure 3c). MiR528 was highly expressed in the embryo at three stages (Figure 3c). The expression level of miR169, miR394 and miR408 in the embryo was the highest at 15 DAP (Figure 3d). MiR168, miR393, miR396 and miR398 were highly expressed in the endosperm at 15 DAP and 20 DAP, indicating their regulation function during middle and late development of the endosperm (Figure 3e).

### 3.3. Differential Expression of miRNAs during Kernel Development

**Figure 4:**
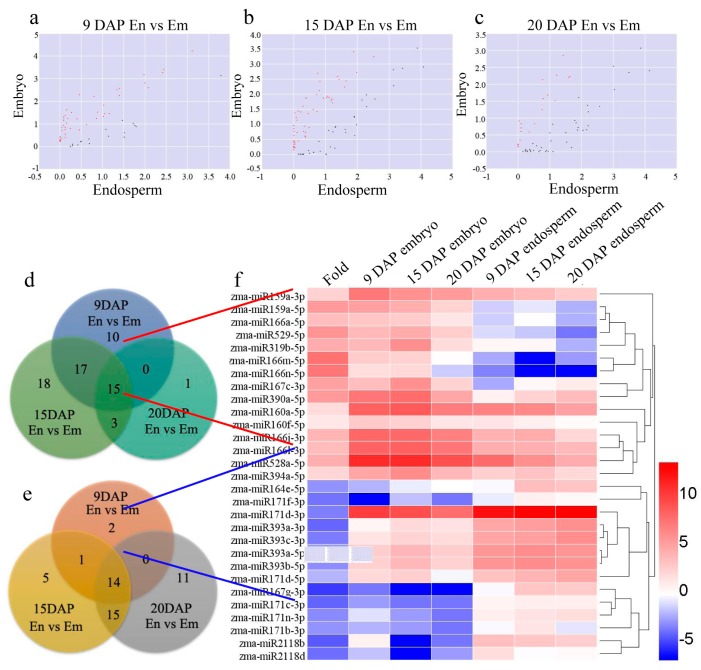
Differentially expressed miRNAs between the embryo and endosperm at three stages. (**a**–**c**) Scatter plots showing differentially expressed miRNAs between the embryo and endosperm at different stages. Red dots mean significantly expressed miRNAs in the embryo compared to the endosperm. Green dots mean significantly expressed miRNAs in the endosperm compared to the embryo. (**d**) Often upregulated expressed miRNAs at different stages when endosperm vs embryo. (**e**) Often downregulated expressed miRNAs at different stages when endosperm vs embryo. (**f**) Hierarchical clustering showing often down- and up-regulated miRNAs among the six tissues.

Differentially expressed miRNAs between the endosperm and embryo were detected across the three stages of kernel differentiation, i.e., endosperm vs embryo (9 DAP), endosperm vs embryo (15 DAP), and endosperm vs embryo (20 DAP) (Figure 4a–c). For kernels at 9 DAP, 42 miRNAs and 17 miRNAs were upregulated and downregulated, respectively (Figure 4d,e). For kernels at 15 DAP, 53 miRNAs and 35 miRNAs were up- and downregulated, respectively (Figure 4d,e). For kernels at 20 DAP, 19 miRNAs and 40 miRNAs were upregulated and downregulated, respectively (Figure 4d,e). All of the differentially expressed miRNAs were statistically significant (*p* < 0.05) with a fold change greater than 2.0. VENN analysis revealed that 15 miRNAs were upregulated and 14 miRNAs were downregulated during all three stages of kernel development (Figure 4d,e). Clusters were generated and analyzed with hierarchical clustering (HCL) for the common differentially regulated miRNAs (Figure 4f). These data suggest that miRNAs are involved in maize kernel development, and that some miRNAs are highly expressed in the endosperm or embryo.

### 3.4. Target of Differentially Expressed Known miRNAs Related to Biosynthetic and Metabolic Process

**Figure 5:**
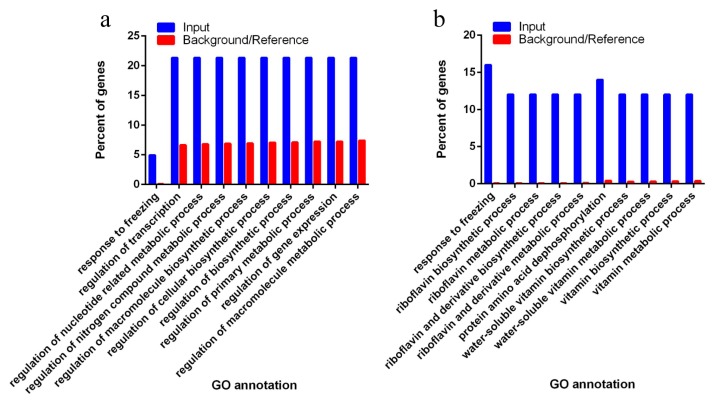
Distributions of differentially expressed miRNA target genes and their functional categories determined by Gene Ontology (GO) analysis. (**a**) Target genes of often highly expressed miRNAs in the embryo. (**b**) Target genes of often highly expressed miRNAs in the endosperm. The *x* axis represents categories of biological functions in different terms. The *y* axis is the percentage of genes involved in a certain function. The blue bars represent the percentage of input genes, targets of the differentially expressed miRNAs between the embryo and endosperm with specific functions. The red bars represent the percentage of the control genomic genes that are involved in the specific function of the same category.

To analyze the function of common differentially expressed miRNAs in maize kernel development, their targets predicated by psRNATarget [43,49] were collected and used for Gene Ontology (GO) enrichment analysis. As a result, 122 and 50 target mRNAs were found and submitted to AgriGO [50], respectively (Tables S5 and S6). After the GO biological process enrichment analysis in contrast to their expression backgrounds/references, these miRNA target genes were functionally categorized into the following specific biological processes with significant differences from their respective backgrounds/references (Figure 5a,b). Target genes of differentially expressed miRNAs in the embryo were largely from “gene regulation including transcription (GO:0045449)”, “gene expression (GO:0010468)”, “macromolecule and cellular biosynthetic progress (GO:0010556; GO:0031326; GO:0009889)”, “macromolecule metabolic process (GO:0019219; GO:0051171; GO:0080090; GO:0060255)” and “response to freezing (GO:0050826)” with significant differences from their backgrounds/references. Target genes of differentially expressed miRNAs in the endosperm were largely from “biosynthetic and metabolic process of riboflavin and vitamin” including “protein amino acid dephosphorylation (GO:GO:0006470; GO:0016311)”, “riboflavin metabolic process (GO:0009231; GO:0006771; GO:0042727; GO:0042726)”, and “vitamin metabolic process (GO:0042364; GO:0006767; GO:0009110; GO:0006766)” and “response to temperature (GO: 0050826; GO:0009409; GO:0009266)” with significant differences from their backgrounds/references.

**Figure 6:**
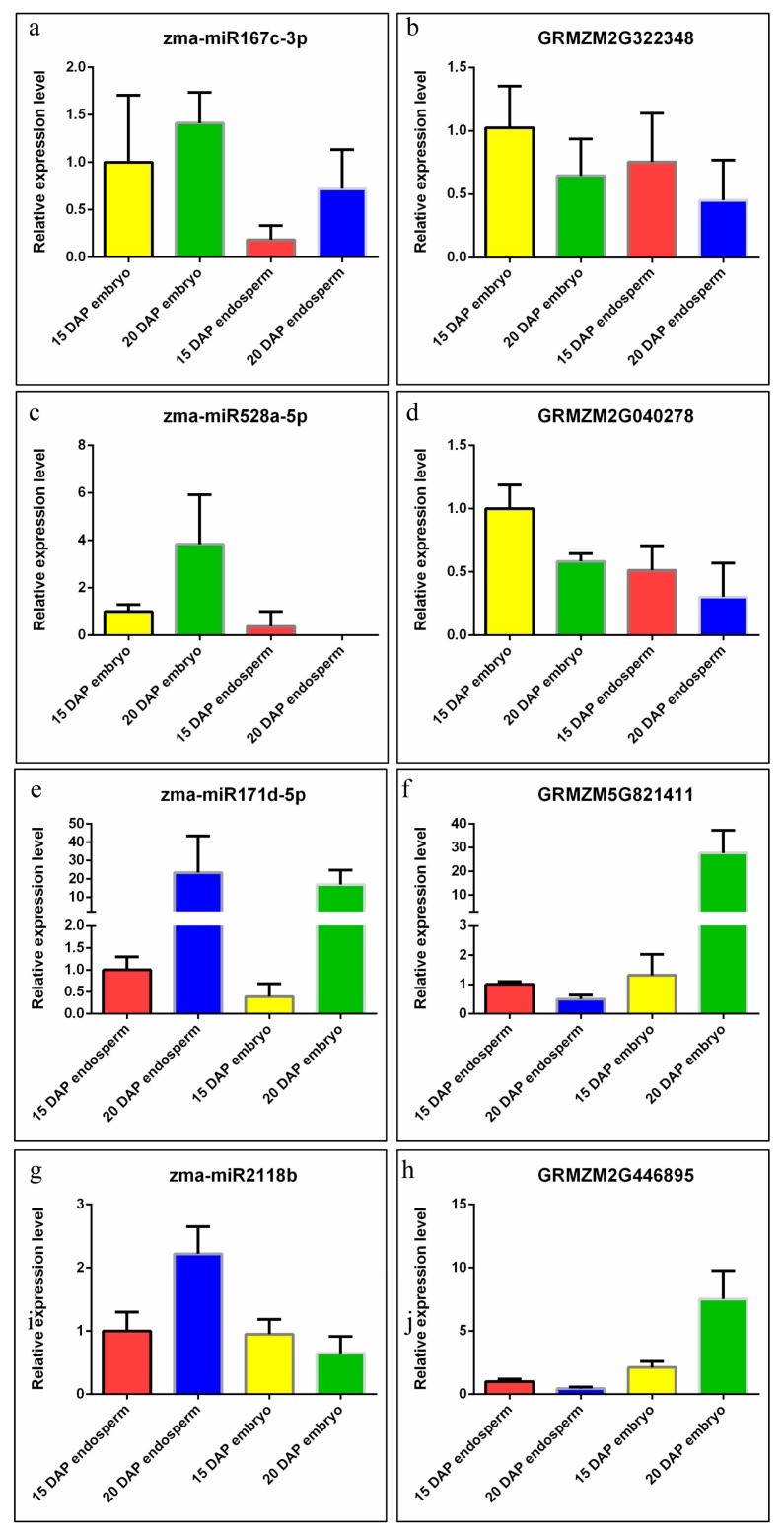
The expression patterns of the embryo-specific and endosperm-specific miRNAs and their target genes at 15 DAP and 20 DAP, respectively. The expression levels of zma-miR167c-3p (**a**), zma-miR528a-5p (**c**), zma-miR171d-5p (**e**), and zma-miR2118b (**g**) were detected by the miRNA quantitative reverse transcription-polymerase chain reaction (qRT-PCR) method. U6 SnRNA of maize was used as an internal control. The expression levels of GRMZM2G322348 (**b**), GRMZM2G040278 (**d**), GRMZM5G821411 (**f**), GRMZM2G446895 (**h**) were detected by the qRT-PCR method. *ACTIN1* of maize was used as an internal control.

To identify the expression patterns of key miRNAs and their targets that are related to maize kernel development, we selected four miRNAs highly expressed in the embryo and endosperm, respectively, including miR167, miR528, miR171, miR2118 and their four targets for further validation by qRT-PCR. As shown in Figure 6, the relative expressions of these miRNA target genes had a strong but simple negative correlation with the levels of their corresponding miRNAs in both the embryo and endosperm during the maize kernel development processes (Figure 6). Meanwhile, zma-miR167c-3p and zma-miR528-5p were expressed more highly in the embryo than the endosperm (Figure 6a,c). Zma-miR171d-5p and zma-miR2118b were expressed more highly in the endosperm than the embryo, except for zma-miR2118b, the expression level of which in the 15 DAP embryo was similar to that in the 15 DAP endosperm (Figure 6e,g).

### 3.5. Zma-miR169 Family Involved in Kernel Development of Maize

**Figure 7:**
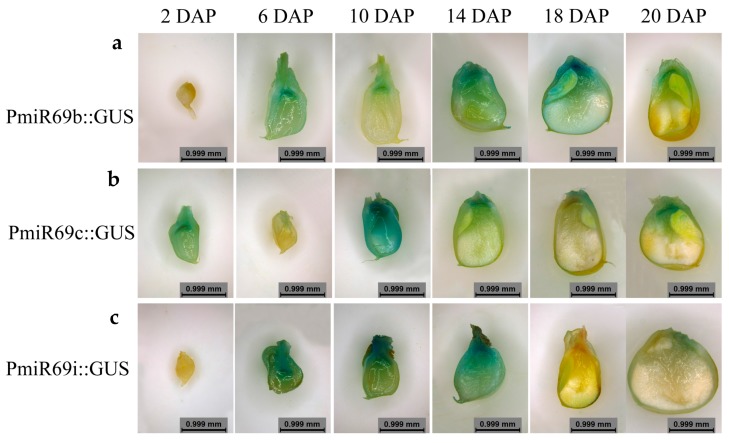
Expression profiles of zma-miR169b (**a**), zma-miR169c (**b**) and zma-miR169i (**c**) in transgenic maize kernels at 2 DAP, 6 DAP, 10 DAP, 14 DAP, 18 DAP and 22 DAP. Plants were planted in a green house with a standard long day (LD) light regime.

The miR169 miRNA family is the largest family and highly conserved in plants. Our previous work found that an miR169 family member plays a key role in stress-induced flowering and leaf development in *Arabidopsis* via regulating its targets genes, NF-YA2 (AT3G05690) and NF-YA10 (AT5G06510) [51,52]. The miR169 family members were screened and identified to be involved in abiotic stress response in maize [53,54]. Here, we found that nine mature miR169 family members were expressed in the embryo or endosperm of maize (Figure 2c). To confirm the sequencing results and investigate the potential biological function of miR169s in maize kernel development, three miR169 family members, zma-miR169b (same mature miRNA sequence as zma-miR169a), zma-miR169c (same as zma-miR169r) and zma-miR169i (same as zma-miR169j and k), were chosen for generating PromiR169::GUS transgenic maize plants and detecting their dynamic expression profiles during kernel development. Through histochemical staining analysis, zma-miR169b, zma-miR169c and zma-miR169i showed varying expression levels in the pedicel, placenta, basal endosperm transfer layer (BETL), embryo and endosperm in their own transgenic kernels (Figure 7a–c), suggesting their important roles in nutrients transport and kernel development. Zma-miR169b was expressed mainly in the endosperm and peaked at 14 DAP (Figure 7a). Zma-miR169c was expressed in the whole kernel with a peak at 10 DAP (Figure 7b). Zma-miR169i was expressed in the whole kernel but peaked at 14 DAP and decreased slowly with kernel development (Figure 7c). Dynamic expression profiles of all the above three miR169 were consistent with the sequencing results (Table S2).

## 4. Discussion

Despite the fact that 321 mature miRNAs from 172 precursors and their expressions at certain specific developmental stages have been reported in maize in the miRBase release 21.0, knowledge of miRNA dynamics in kernel development and specifically expressed in the embryo and endosperm is scarce. In this study, a high-throughput sequencing method was employed for investigating the miRNA dynamic profiles in the early stage and reserve substance filling during the development of the embryo and endosperm. We collected the samples at three stages: 9 DAP, 15 DAP and 20 DAP. The kernel at 9 DAP represents the differentiation peaks of cell types in the embryo and endosperm. The kernel at 15 DAP represents the transition of the kernel from cellularization to the filling stage. The kernel at 20 DAP represents the programmed cell death (PCD) initiation stage. Via high-throughput sequencing and qRT-PCR inspection, we discovered the dynamic profiles of miRNAs in maize kernel during early and middle development phases. Remarkably, we found that miR169 might be a kernel-specific miRNA, involved in the regulation development of the embryo or endosperm.

### 4.1. Differential and Specific Expression Patterns of miRNAs May Determine the Differential Kernel Development Patterns of the Embryo and Endosperm

In this study, we identified 132 known miRNAs and six novel miRNAs in at least one of the six sRNA libraries at three kernel developmental stages in maize. The expression patterns of miRNAs display a developmental stage-dependent and/or tissue-specific style during the maize kernel developmental process in this study. Similar results were found during the grain filling process in superior and inferior spikelets of rice [11]. The expression levels of most miRNAs increased gradually with the early kernel development in both the embryo and endosperm (Figure 2a, Table S2). MiRNAs in the embryo were more highly expressed at the early and middle stage (9 and 15 DAP), peaked at 15 DAP, and decreased at 20 DAP. However, in the endosperm, most miRNAs had higher expression levels at the filling stage (20 DAP) (Figure 2a, Table S2). We speculated that the tissue-specific and differential expression patterns of miRNAs determined the developmental differences between the embryo and endosperm in maize.

There are 14 miRNA families displaying significantly different expression patterns between the embryo and endosperm. Although miR159a-3p and miR159a-5p maintained good amounts both in the embryo and endosperm, they were expressed significantly higher in the embryo in our study (Table S2). It has been reported that miR159 was expressed higher in inferior spikelets than in superior spikelets in rice [11]. Moreover, miR159ab double mutant has been reported to have pleiotropic morphological defects, including altered growth habit, curled leaves, small siliques and small kernels in *Arabidopsis* [55]. Another individual miRNA, miR166, was specifically expressed in the maize embryo at three stages, consistent with results in barely [47]. It has been reported that miR166 positively controls the leaf polarity through downregulating its target, *rld1*, a class III homeodomain/leucine zipper TF in maize [56]. The MiR166/HD-ZIP III module participated in regulating cell differentiation in root and shoot apical meristems of *Arabidopsis* [57,58]. MiR166-targeted transcription factor gene *rice Dof daily fluctuations 1* (*RDD1*) contributes to the increased grain productivity of rice via inducing the efficient uptake and accumulation of various nutrient ions [59]. These findings indicate that miR166-mediated repression of transcription factors might be conserved for modulating cell differentiation during kernel development. Meanwhile, lower and dynamic expression of five miR166 members during the developmental process of endosperm might be involved in the reserve filling (Figure 2a, Table S2). Another conserved miRNA family, miR393, was found to accumulate more in the endosperm than in the embryo through three stages. MiR393-targeted *transport inhibitor response protein 1* (TIR1) and *auxin signaling F-box protein* (*AFBs*) affect seed development in barley and nitrogen-promoted tillering in rice through the regulation of auxin signaling [47,60].

### 4.2. miR169 Family Members Specifically Were Expressed in Maize Kernel

As the most conserved and largest miRNA family in maize, miR169 has 18 members and can be divided into the following 10 subgroups based on mature miRNA sequences: miR169a/b, miR169c/r, miR169f/g/h, miR169i/j/k, miR169d, miR169e, miR169l, miR169o, miR169p and miR169q/n/m. MiR169 was defined as stress-responsive miRNA because its family members are largely involved in plant responses to abiotic stress [61,62,63,64,65,66,67,68,69]. Moreover, recent studies have proved that miR169 family members were involved in complex regulatory networks by coordinating stress responses and various developmental processes instead of working independently. For example, miR169d was involved in flowering signaling networks and regulated stress-induced early flowering in *Arabidopsis* [51]. *Nuclear factor Y subunit A* (*NF-YAs*), targets of miR169, acted as a negative regulator in rice immunity against the blast fungus *Magnaporthe oryzae* in rice [70]. MiR169 may play important roles during seed germination under salt- or drought-stress conditions in *Brassica napus* [71]. In our previous work, we found that zma-miR169 family members responded to the three abiotic stress conditions polyethylene glycol (PEG), abscisic acid (ABA) and NaCl, and most of the targeted ZmNF-YA genes exhibited a reverse correlation with zma-miR169 gene expression [53]. In this work, we identified nine mature miR169 members that were expressed in at least one sample via high-throughput sequencing. We also demonstrated that miR169 affected kernel development through a promoter: the GUS system in transgenic maize plants (Figure 7), although the detailed mechanism and phenotype changes need further elucidation.

## Supplementary Materials

The following are available online at www.mdpi.com/2073-4425/8/12/385/s1. Figure S1: Secondary structures of precursor of six novel maize miRNAs, Table S1: List of primers used in this study, Table S2: Characteristics of all known miRNAs identified in this study, Table S3: Novel miRNAs predicted by miRDeep2, Table S4: Known miRNAs were highly expressed in this study (TPM > 50), Table S5: Target genes of embryo- and endosperm-specific miRNAs, Table S6: GO analysis for target genes of embryo- and endosperm-specific miRNAs.
