# Supplementary figures and images for: High-Throughput Sequencing of Small RNA Transcriptomes in Maize Kernel Identifies miRNAs Involved in Embryo and Endosperm Development
Source: Genes (Basel). 2017 Dec 14;8(12):385. doi: 10.3390/genes8120385 (PMC5748703; doi:10.3390/genes8120385)

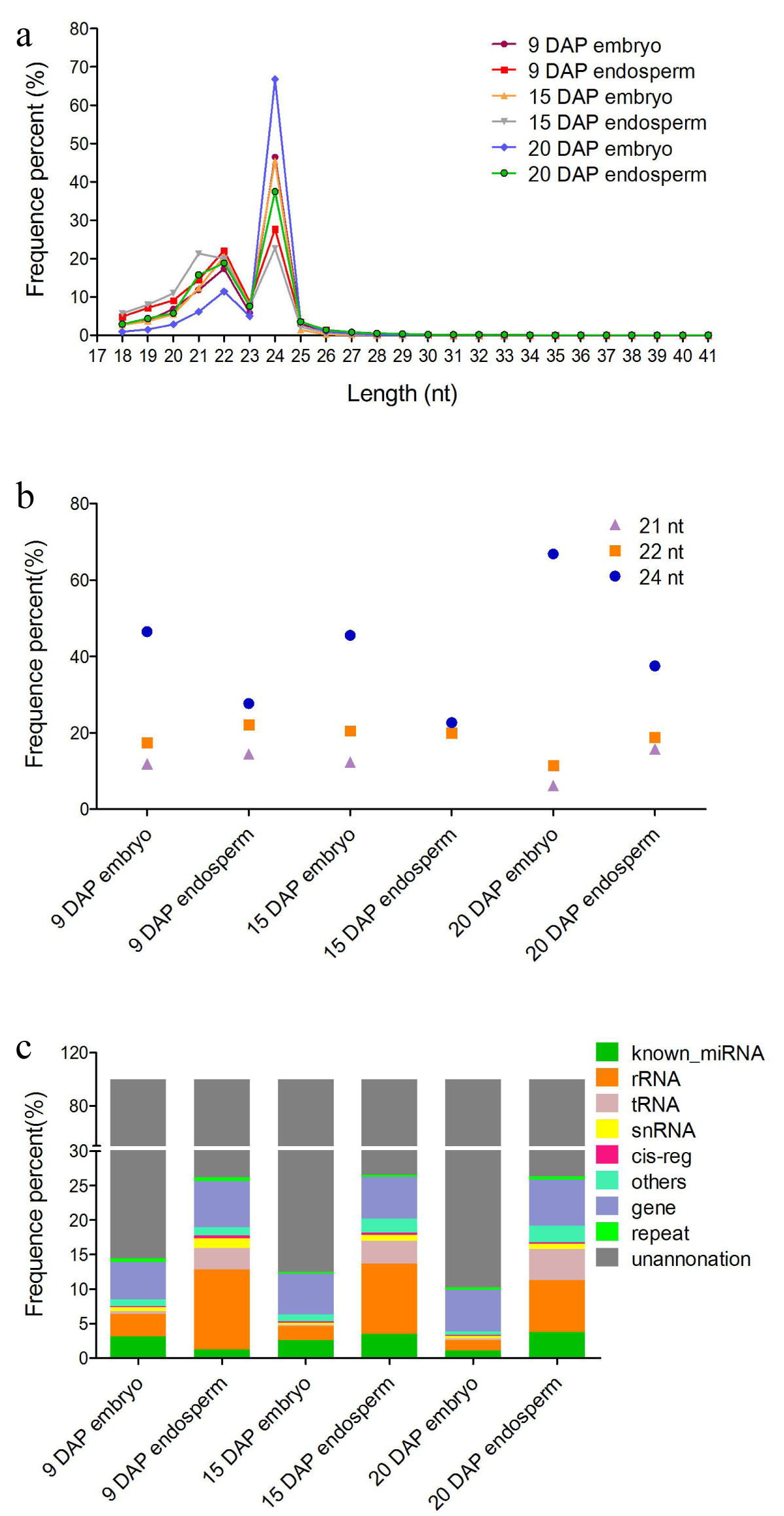

Supplement: Supplementary file 1 [file genes-08-00385-s001.zip › Figure1-7/Figure 1.tif]

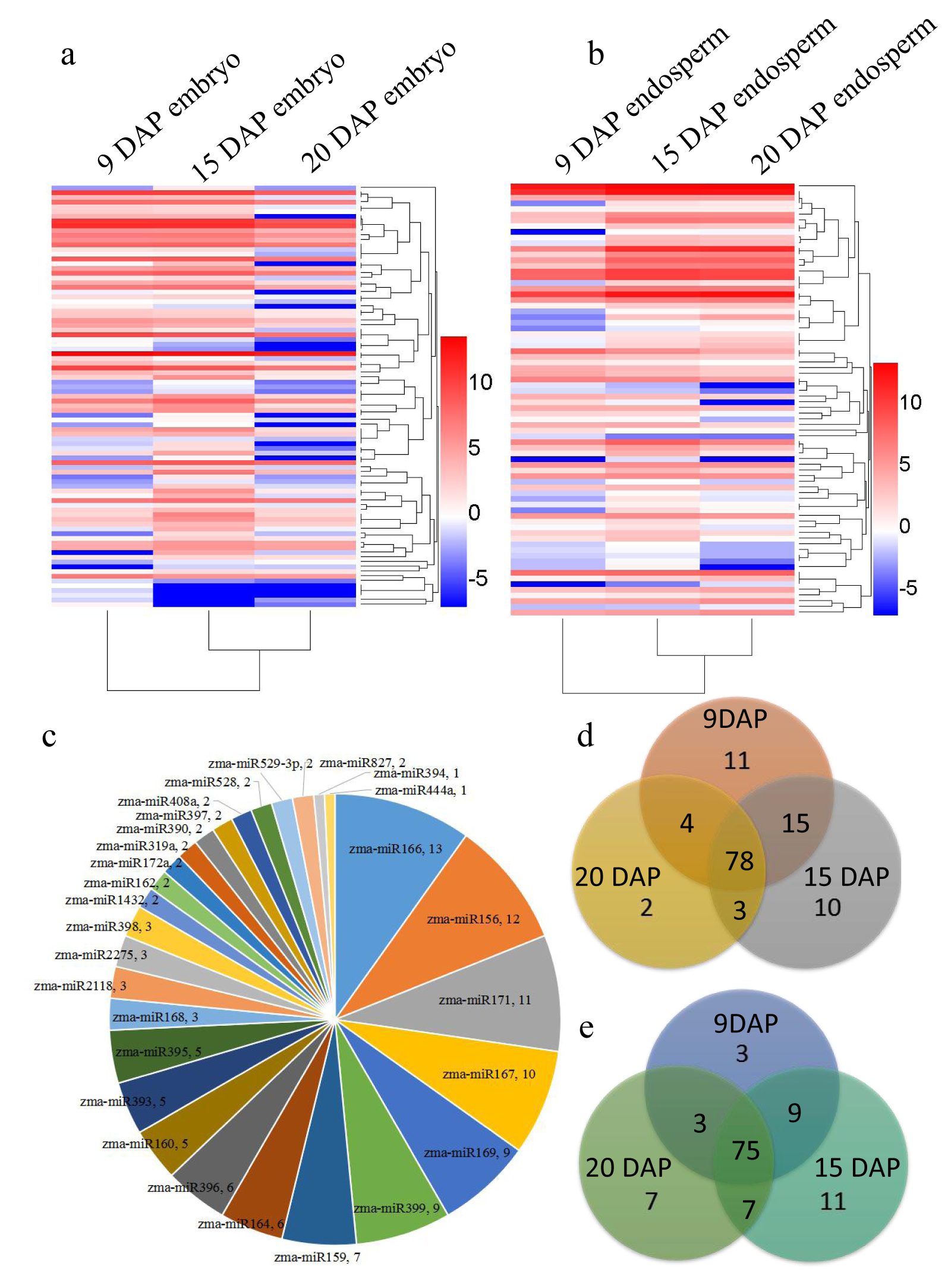

Supplement: Supplementary file 1 [file genes-08-00385-s001.zip › Figure1-7/Figure 2.tif]

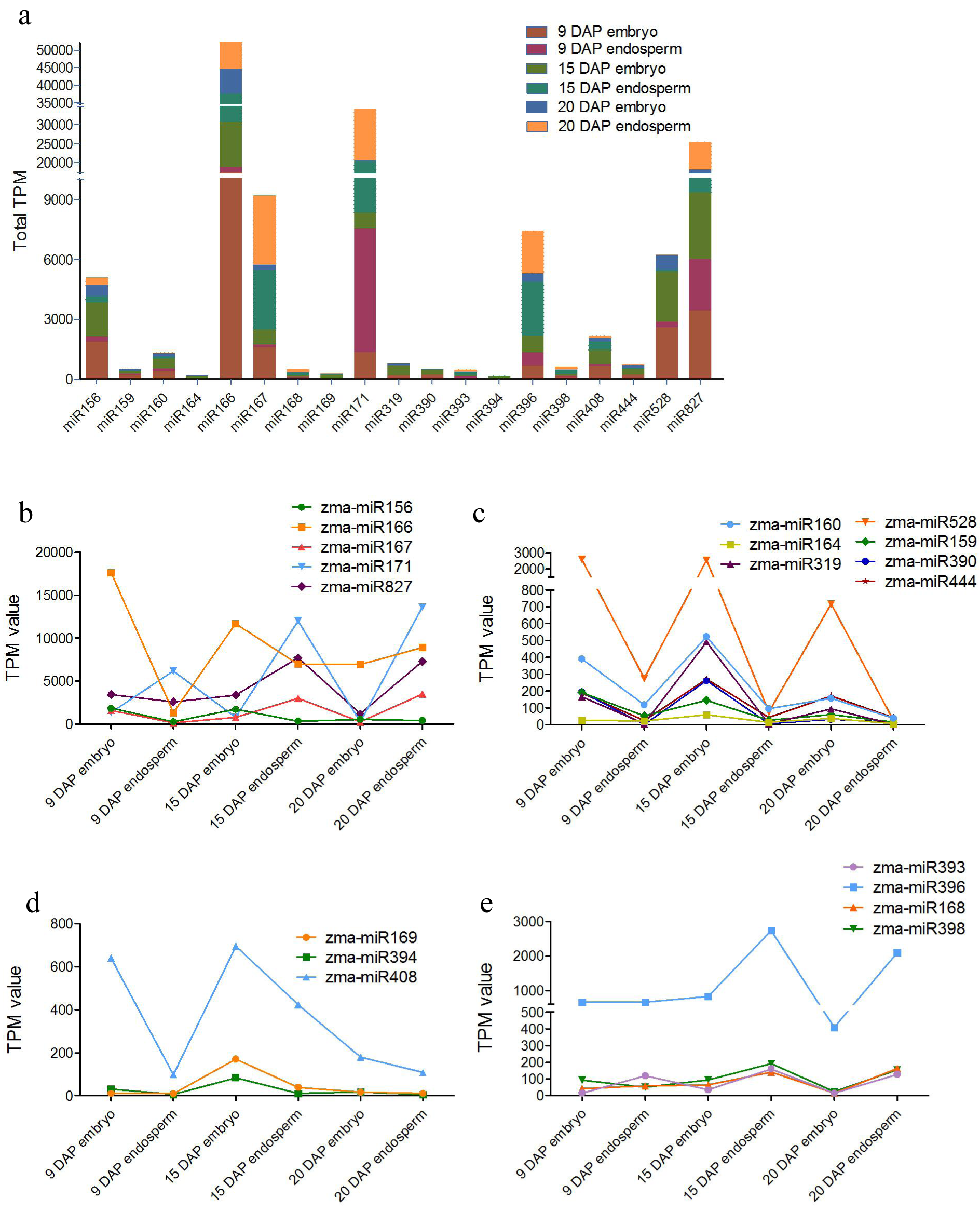

Supplement: Supplementary file 1 [file genes-08-00385-s001.zip › Figure1-7/Figure 3.tif]

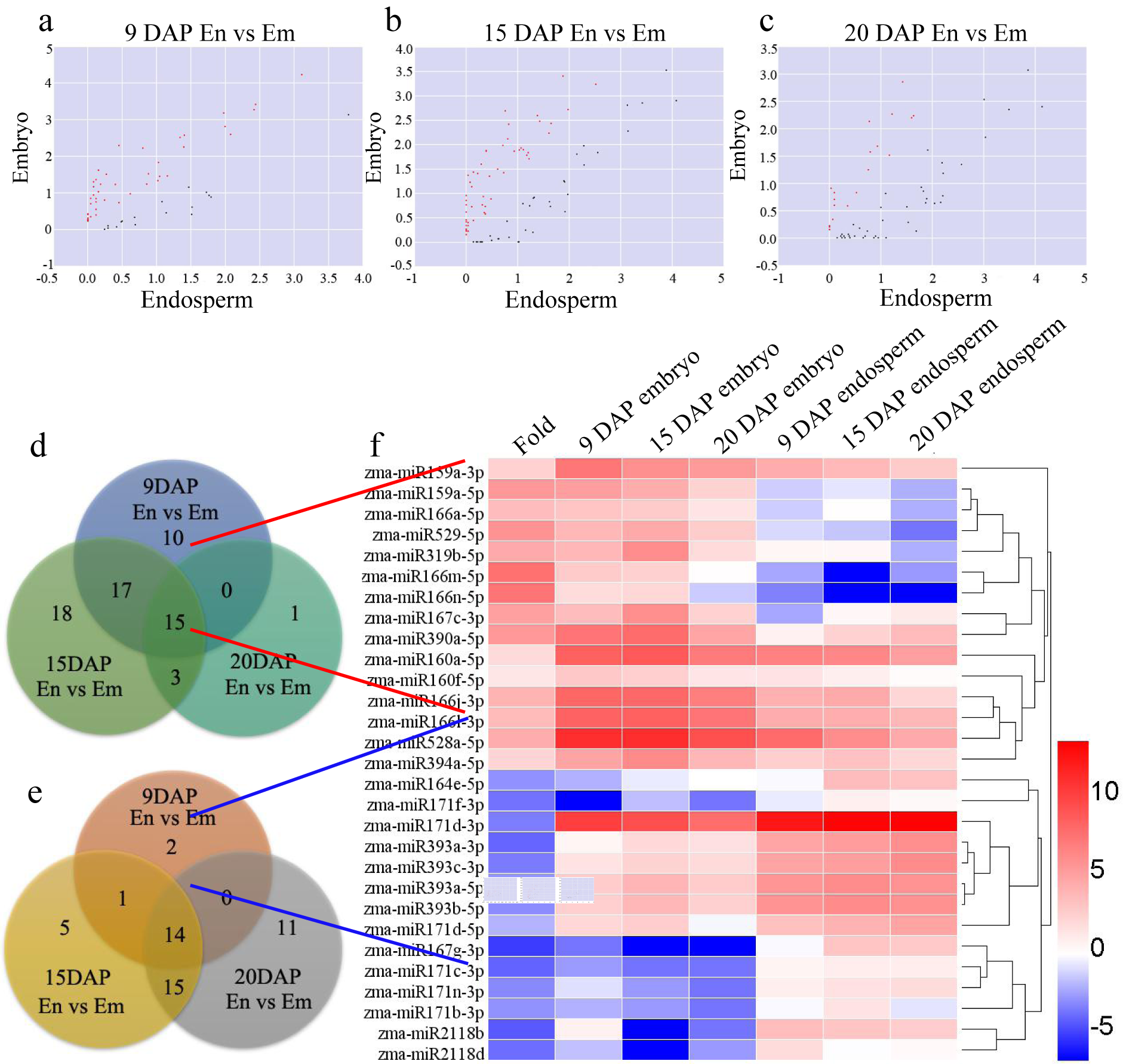

Supplement: Supplementary file 1 [file genes-08-00385-s001.zip › Figure1-7/Figure 4.tif]

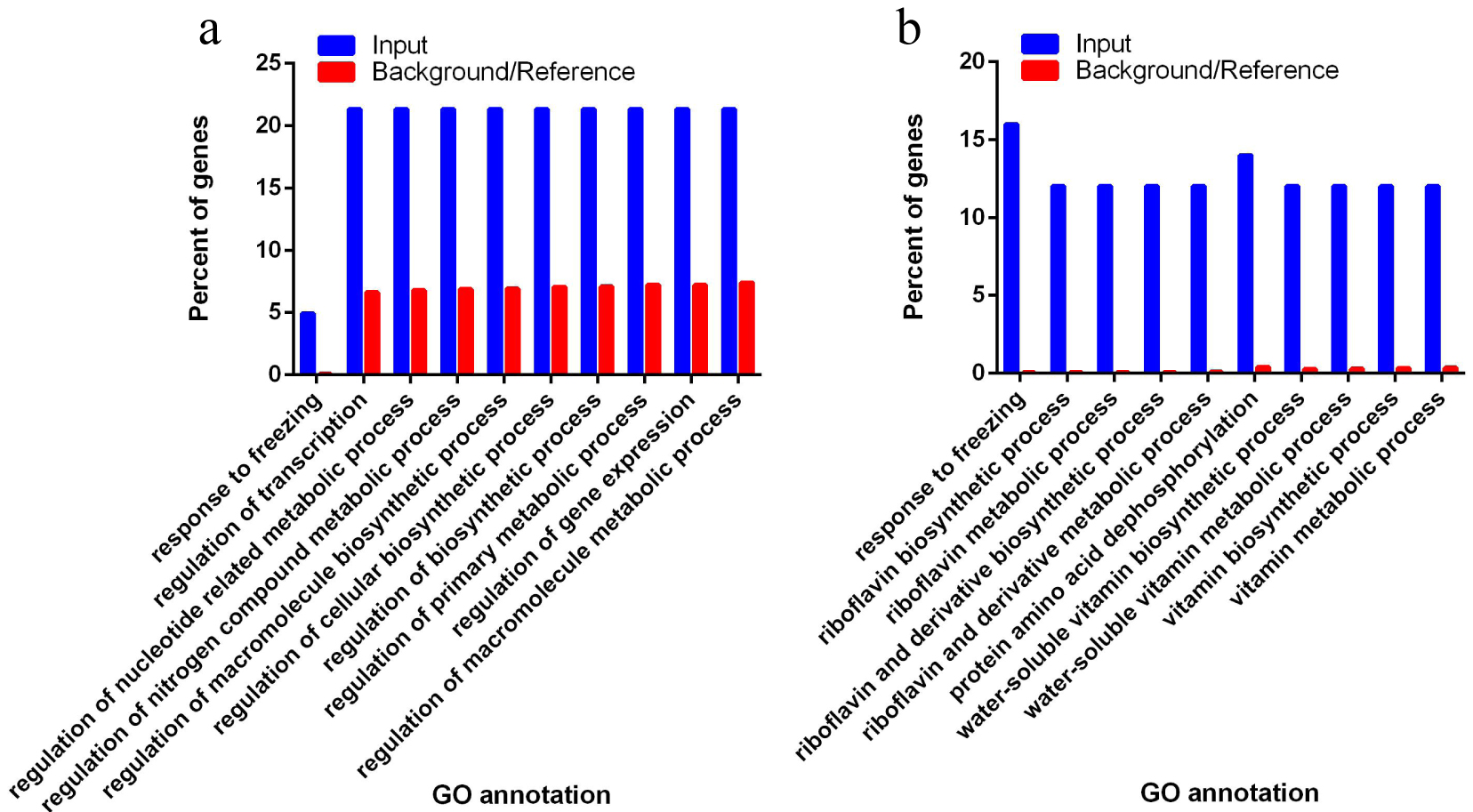

Supplement: Supplementary file 1 [file genes-08-00385-s001.zip › Figure1-7/Figure 5.tif]

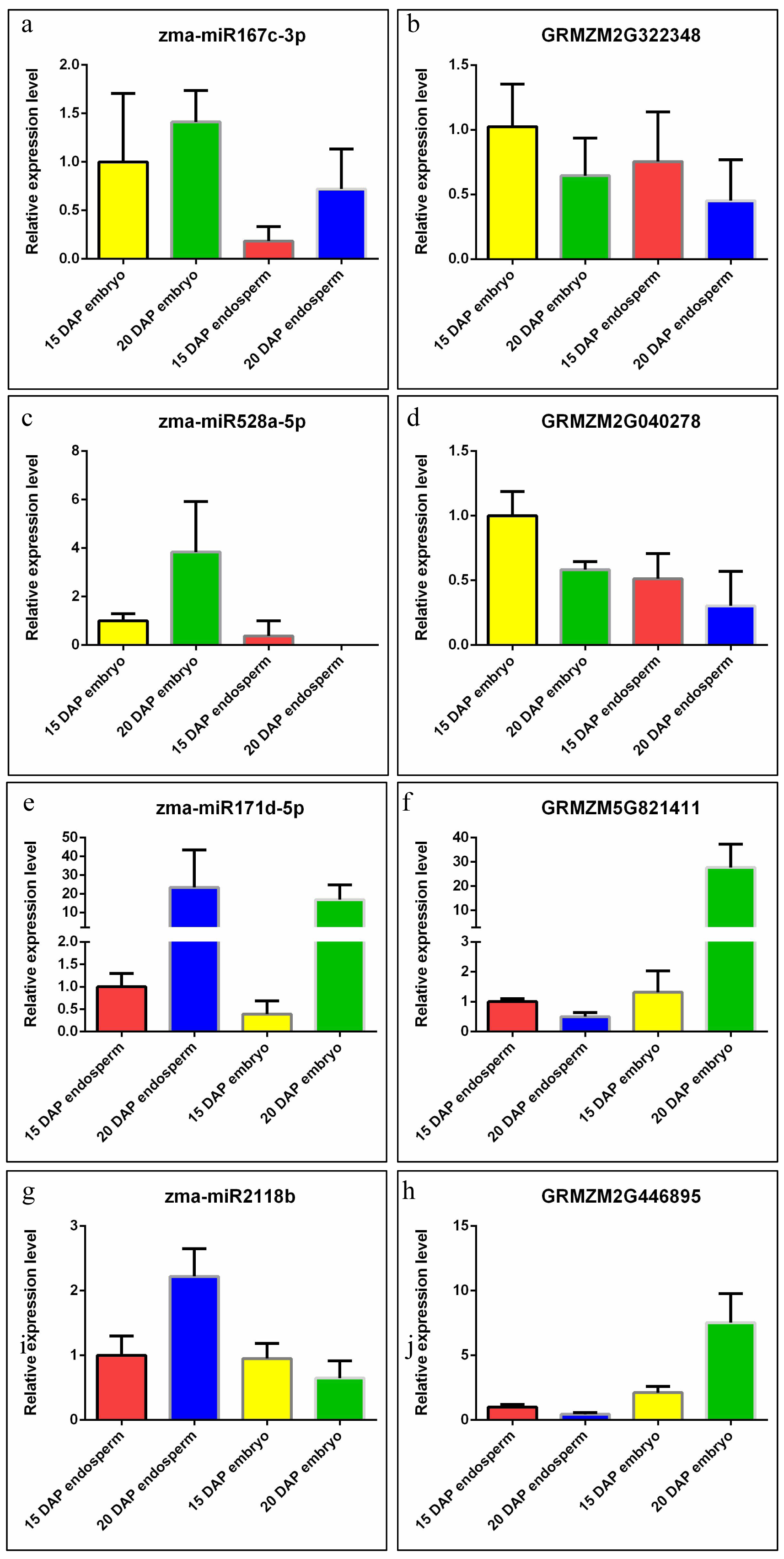

Supplement: Supplementary file 1 [file genes-08-00385-s001.zip › Figure1-7/Figure 6.tif]

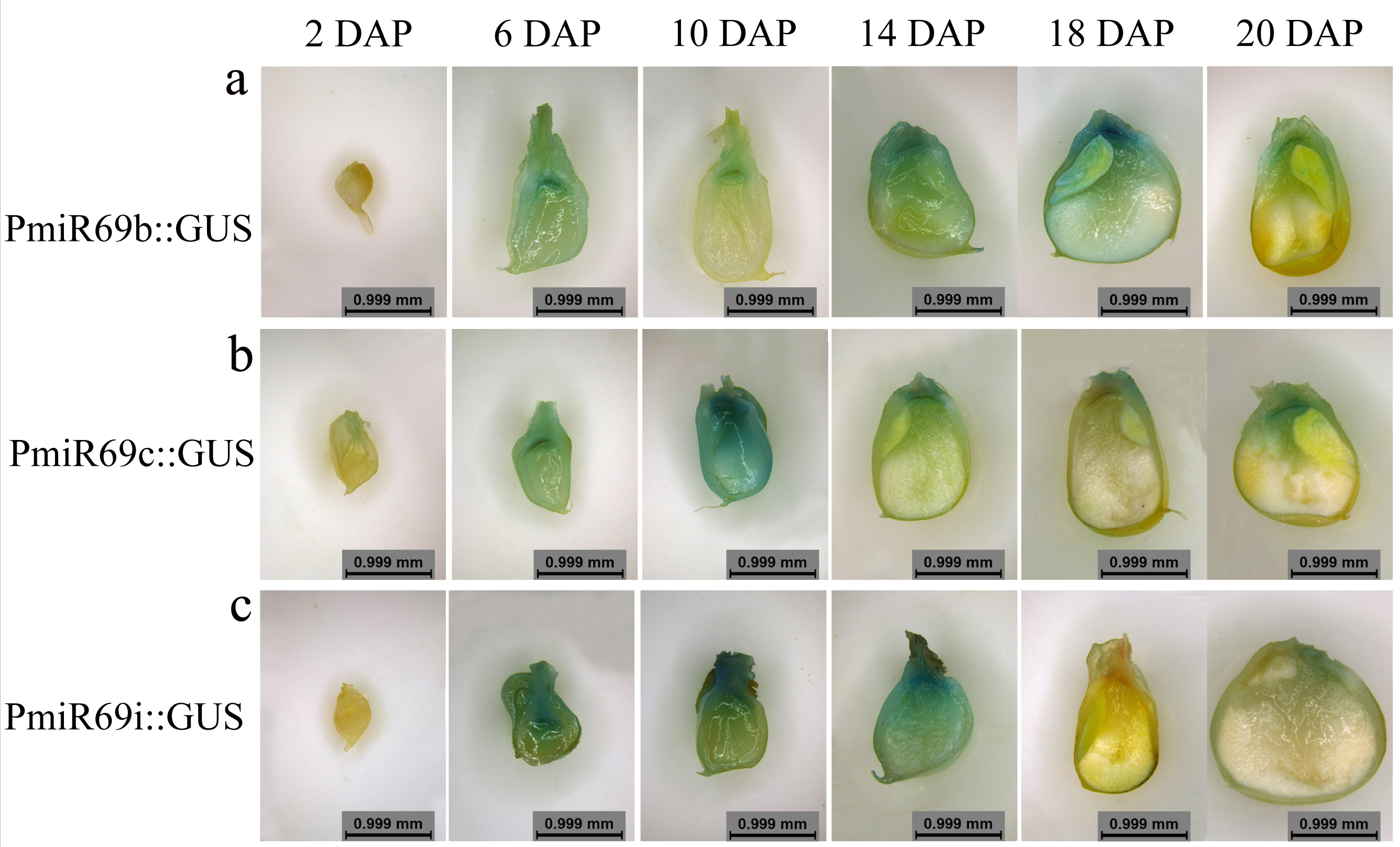

Supplement: Supplementary file 1 [file genes-08-00385-s001.zip › Figure1-7/Figure 7.tif]
